# Correlation Between Platelet Indices and Severity of Sepsis: A Hospital-Based Prospective Study

**DOI:** 10.7759/cureus.82816

**Published:** 2025-04-22

**Authors:** Krishna Padarabinda Tripathy, Yelisetti Chaitanya, Pradip K Behera, Ranjita Panigrahi, Devi P Dash

**Affiliations:** 1 General Medicine, Kalinga Institute of Medical Sciences, Kalinga Institute of Industrial Technology (KIIT) University, Bhubaneswar, IND; 2 Pathology, Kalinga Institute of Medical Sciences, Kalinga Institute of Industrial Technology (KIIT) University, Bhubaneswar, IND; 3 Community and Family Medicine, Kalinga Institute of Medical Sciences, Kalinga Institute of Industrial Technology (KIIT) University, Bhubaneswar, IND

**Keywords:** mean platelet volume (mpv), plateletcrit, platelet distribution width (pdw), platelet large cell ratio (p-lcr), sofa and apache ii

## Abstract

Background: Sepsis is a life-threatening condition that causes tissue and organ damage, with older adults, very young children, pregnant women, and individuals with comorbidities at higher risk. This study evaluates the correlation between platelet indices and the severity of sepsis by comparing them with the Sequential Organ Failure Assessment (SOFA) and Acute Physiology and Chronic Health Evaluation (APACHE II) scores, aiming to establish cost-effective and easily accessible hematological prognostic markers for sepsis management.

Methods: A prospective study was conducted involving 320 patients with sepsis to evaluate platelet indices, such as mean platelet volume (MPV), platelet distribution width (PDW), plateletcrit (PCT), and platelet large cell ratio (P-LCR). The association between these indices and sepsis outcomes was analyzed. Platelet indices were compared with the gold-standard SOFA and APACHE II scores. Statistical analysis was applied to both categorical and continuous data, while a t-test was applied for comparison. Correlation analysis was performed using the Pearson correlation coefficient. P-values less than 0.05 were considered statistically significant.

Results: The clinico-demographic profile of sepsis patients revealed a mean age of 55.17 ± 18.10 years, with 67% male patients. Platelet indices such as PDW and PCT were elevated on day 3 and showed comparability with SOFA scores. A significant association was found between clinico-demographic features and sepsis outcomes (death and discharge). Among the platelet indices, MPV on days 1 and 7, PDW on days 3 and 7, P-LCR on days 3 and 7, and PCT on day 7 showed significant associations with sepsis outcomes. In correlation analysis, PDW on days 3 and 7 and PCT on day 7 demonstrated a significant positive correlation with SOFA and APACHE II scores. Conversely, a significant negative correlation was found between PCV and the SOFA score.

Conclusions: The study concludes that PDW and PCT on days 3 and 7 are strong predictive markers of sepsis severity, while P-LCR on day 7 is effective in predicting sepsis outcomes. Overall, platelet indices offer valuable prognostic insight for assessing sepsis severity and outcomes, especially in resource-limited hospital settings.

## Introduction

Sepsis is a serious and life-threatening condition that leads to organ dysfunction, with a 20% fatality rate [1,2]. Vulnerable populations are at greater risk of developing sepsis. Clinical signs and symptoms include low body temperature or fever, altered sensorium, tachycardia, low-volume pulse, low blood pressure, tachypnea, decreased urine output, cyanosed mucous membranes, and cold extremities [3,4]. Post-sepsis syndrome is associated with long-term mortality, a heightened risk of infections, and organ dysfunction across cognitive, renal, cardiovascular, and psychological domains compared to non-sepsis patients [5].

Early recognition and timely intervention are critical in the management of sepsis to improve outcomes. A lack of hemostatic balance and endothelial dysfunction significantly affect the circulatory system and intracellular homeostasis. Cellular hypoxia and apoptosis, along with the release of cytokines (such as tumor necrosis factor-alpha, interleukins, and prostaglandins), and the activation of neutrophils and macrophages contribute to organ malfunction and mortality. Activation of the extrinsic coagulation cascade, impaired fibrinolysis, and microvascular thrombosis are also key contributors to organ failure in sepsis [5]. Currently, the severity of sepsis is assessed using scoring systems such as the Sequential Organ Failure Assessment (SOFA) and Acute Physiology and Chronic Health Evaluation (APACHE II) [5]. Biomarkers including C-reactive protein, serum procalcitonin, serum lactate, presepsin (sCD14), and interleukin-6 are used for early diagnosis and prognostication. However, these markers are less effective in predicting mortality when used individually and are often unaffordable in low-resource settings [6]. Platelets play a crucial role in hemostasis by forming a platelet plug at sites of endothelial injury. They are also involved in physiological and pathological processes such as inflammation, coagulation, and the regulation of vascular endothelial cell integrity. In sepsis, alterations in the coagulation cascade are reflected in prolonged activated partial thromboplastin time (aPTT), prothrombin time (PT), and reduced platelet count. Platelet parameters are available as part of complete blood count (CBC) results [7]. Mean platelet volume (MPV) reflects platelet size, while platelet distribution width (PDW) is a marker with high specificity for platelet activation [8]. Plateletcrit (PCT) is useful in detecting quantitative abnormalities in platelets and serves as a reliable screening tool [9]. Given their inclusion in standard CBCs and their affordability compared to advanced biomarkers, platelet indices may serve as valuable predictive markers for sepsis severity. These indices have already shown prognostic relevance in conditions such as acute appendicitis, pancreatitis, infective endocarditis, and malaria [10]. Thus, the present study aims to evaluate the correlation between platelet indices and the gold-standard SOFA and APACHE II scores at different time points, in order to determine their predictive value in assessing sepsis severity.

## Materials and methods

Study design and setting 

A prospective observational study was conducted at the Department of Medicine, Pradyumna Bal Memorial Hospital, Kalinga Institute of Medical Sciences, KIIT University, for 18 months. A written consent was obtained from all participants prior to enrolment in the study. 

Population

All consecutive patients diagnosed with sepsis and admitted to the ICU (with a SOFA score > 2) during the 18-month study period who met the inclusion criteria were enrolled as the study population. A convenient sampling method was used to achieve a sample size of 320 patients.

Inclusion and exclusion criteria 

Inclusion criteria included patients aged over 18 years who were diagnosed with sepsis and had a SOFA score greater than 2. Exclusion criteria were patients with bone marrow disorders, viral infections, autoimmune conditions, and those undergoing chemotherapy, radiation therapy, or treatment with antiepileptic drugs, immunosuppressants, or antiplatelet agents. Patients with hypersplenism or those who discontinued treatment were also excluded.

Study tools

Basic demographic information, comorbidities, and the source of infection, along with presenting vital signs, were recorded. Blood samples were collected and analyzed for CBC with differential, red blood cell (RBC) indices, platelet indices, blood urea, serum creatinine, serum electrolytes, liver function test (LFT), coagulation parameters (international normalized ratio (INR), aPTT), serum glucose, lactate levels, CRP, serum procalcitonin, and cultures (blood and others). Arterial blood gas (ABG) analysis was performed using an autoanalyzer (XN-1000, Sysmex Corporation, Kobe, Japan). Chest X-ray and ECG were also conducted to assess clinical manifestation.

The SOFA scoring system was used to assess six major organ systems (respiratory, cardiovascular, hepatic, renal, neurological, and coagulation), with scores ranging from 0 to 4 for each system. Sepsis was defined as an increase of two or more points in the SOFA score.

The APACHE II scoring system was employed as a predictive tool to assess disease severity by using a combination of 12 routine physiological parameters (such as temperature, heart rate, blood pressure, and mean arterial pressure), hematological parameters, age, and the Glasgow Coma Scale. The total score ranges from 0 to 71, with scores above 17 indicating a higher risk of mortality. 

SOFA and APACHE II scores were calculated for all sepsis patients on days 1, 3, and 7 of ICU admission. Platelet indices were also measured at ICU admission and on days 1, 3, and 7.

Statistical analysis

All continuous and categorical variables were analyzed using IBM SPSS Statistics for Windows, Version 27.0 (Released 2020; IBM Corp., Armonk, NY, United States). Data were presented as mean ± standard deviation, or median with interquartile range (IQR), depending on the distribution. Comparisons of platelet indices with SOFA and APACHE II scores were performed using the Kruskal-Wallis test and Spearman’s rank correlation. One-way analysis of variance (ANOVA) was used to compare means where appropriate. A p-value less than 0.05 was considered statistically significant.

Ethical consideration 

The study was approved by the Institutional Ethics Committee (KIIT/KIMS/ IEC/126/2019) prior to patient enrolment.

## Results

A total of 320 patients diagnosed with sepsis were included in the study. The mean age of the cohort was 55.17 years ± 18.10 years. Clinico-demographic analysis highlighted differences in sepsis outcomes across various age groups.

**Figure 1:**
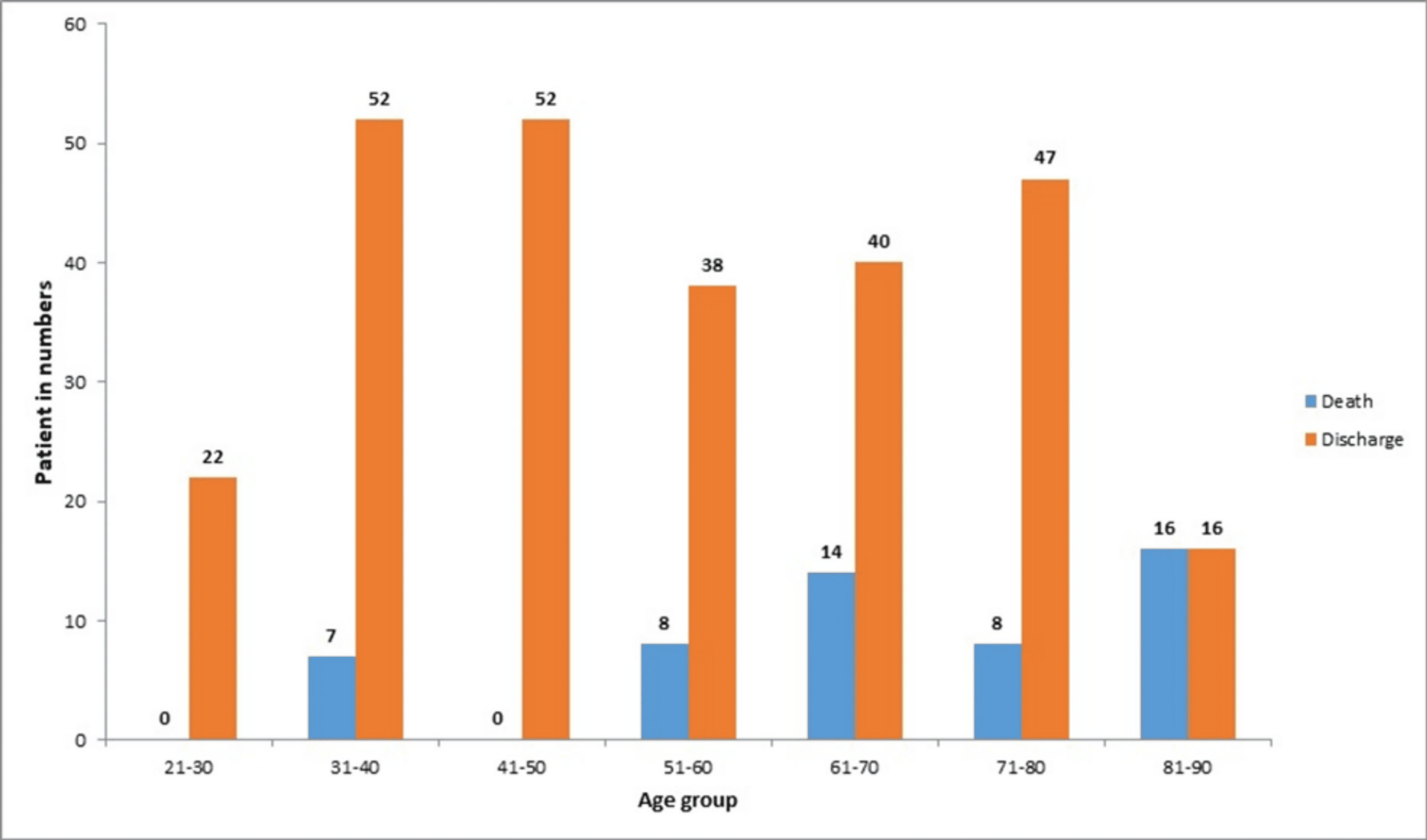
Overview of sepsis outcomes among different age groups.

Among the 53 patients who died, the majority were over 60 years of age. In contrast, among the 267 discharged patients, the highest proportion belonged to the 30-50 age group. A chi-square test demonstrated a strong association between age and sepsis outcome (chi-square, 45.1321; p < 0.0001) (Figure 1).

In terms of gender distribution, male patients comprised a higher proportion (67.2%) compared to female patients (32.8%). The severity of sepsis was assessed by SOFA and APACHE II scoring systems on days 1, 3, and 7. On days 1 and 3, the mean SOFA score ranged between 5 and 6, while on day 7, it decreased to 4.8. The mean APACHE II score was highest on day 1 (17.88 ± 6.56) compared to days 3 and 7. Regarding clinical outcomes, of the 320 patients, 53 did not survive, while the remaining 267 (83.4%) recovered and were discharged (Table 1).

**Table 1:** Clinico-demographic features of patients with sepsis. SOFA: Sequential Organ Failure Assessment, APACHE II: Acute Physiology and Chronic Health Evaluation.

Characteristics	N = 320
Age (mean ± SD)	55.17 ± 18.10
Gender	
Male	215 (67%)
Female	105 (33%)
Predictive score	
SOFA	
Day 1 (mean ± SD)	5.26 ± 2.05
Day 3 (mean ± SD)	5.6 ± 2.4
Day 7 (mean ± SD)	3.1 ± 3.1
APACHE II	
Day 1 (mean ± SD)	16.88 ± 6.5
Day 3 (mean ± SD)	13.83 ± 5.8
Day 7 (mean ± SD)	12.37 ± 5.8
Clinical outcome	
Death	53 (17%)
Discharge	267 (83%)

Among platelet indices, the MPV was 11.35 ± 1.48 on day 1, followed by 11.54 ± 1.44 on day 3, and 11.33 ± 1.53 on 7. The PDW was 15.67 ± 4.00 on day 1 and 16.27 ± 3.97 on days 3 and 7. The mean P-LCR ranged between 38% and 40% across all three days, which was slightly above the normal value (15%-35%). The mean PCT was 0.5142 ± 1.7409 on day 1, 0.22 ± 0.12 on day 3, and 0.22 ± 0.14 on day 7 (Table 2).

**Table 2:** Platelet indices on days 1, 3, and 7 of patients with sepsis.

Platelet indices	Mean ± SD
Mean platelet volume (MPV)	
Day 1	11.35 ± 1.48
Day 3	11.54 ± 1.44
Day 7	11.33 ± 1.53
Platelet distribution width (PDW)	
Day 1	15.67 ± 4.00
Day 3	16.27 ± 3.9
Day 7	15.46 ± 4.0
Platelet large cell ratio (P-LCR)	
Day 1	39.83 ± 8.39
Day 3	40.60 ± 9.63
Day 7	38.27 ± 9.54
Plateletcrit (PCT)	
Day 1	0.514 ± 1.74
Day 3	0.22 ± 0.12
Day 7	0.22 ± 0.14

Sepsis outcome and prediction scores 

In terms of sepsis outcomes, mortality was higher among female patients, with 29 deaths (54.7%), compared to 24 deaths (45.3%) among male patients. Among those who died, the mean age was 66.22 ± 15.38 years, whereas the mean age of discharged patients was 52.98 ± 17.82 years (Table 3).

**Table 3:** A significant prediction of sepsis outcomes observed based on age and SOFA and APACHE II scores. SOFA: Sequential Organ Failure Assessment, APACHE II: Acute Physiology and Chronic Health Evaluation. *Significant at p < 0.05.

Clinical features	Death (n = 53; mean ± SD)	Discharge (n = 264; mean ± SD)	t-value	p-value
Age (in years)	66.22 ± 15.38	52.98 ± 17.82	5.04	0.0001*
SOFA score				
Day 1	5.56 ± 2.91	5.20 ± 1.84	1.17	0.239
Day 3	7.01 ± 2.58	5.39 ± 2.32	4.56	0.0001*
Day 7	6.94 ± 3.46	4.36 ± 2.96	5.62	0.0001*
APACHE II score				
Day 1	18.35 ± 3.16	16.98 ± 7.04	0.63	0.0001*
Day 3	14.86 ± 7.01	12.98 ± 4.58	1.17	0.0058*
Day 7	14.00 ± 6.02	12.81 ± 6.25	0.59	0.0001*

For severity prediction in sepsis, the mean SOFA score on day 1 was 5.56 ± 2.91, and the mean APACHE II score was 18.35 ± 3.16. In contrast, among patients who were discharged, the mean SOFA score was 5.20 ± 1.84, and the mean APACHE II score was 16.98 ± 7.04. When comparing scores between the death and discharge groups, on day 1, a significant difference was observed in the APACHE II score (p = 0.0001), but on days 3 and 7, both SOFA and APACHE scores were found to significantly predict disease severity. 

Association of platelet indices with sepsis outcomes

The association between platelet indices and sepsis outcome (death and discharge) is displayed in Table 4.

**Table 4:** Association between platelet indices and sepsis outcomes. *Significant at p < 0.05.

Platelet indices	Death	Discharge	t-value	p-value
Mean platelet volume				
Day 1	12.08 ± 0.84	11.20 ± 1.54	4.07	0.0001*
Day 3	12.04 ± 1.39	11.44 ± 1.43	2.77	0.0058*
Day 7	12.12 ± 1.38	11.17 ± 1.52	4.17	0.0001*
Platelet distribution width				
Day 1	16.18 ± 1.96	15.57 ± 4.29	1.01	0.3086
Day 3	18.93 ± 3.64	15.74 ± 3.83	5.58	0.0001*
Day 7	18.65 ± 4.00	14.83 ± 3.70	6.76	0.0001*
Platelet large cell ratio				
Day 1	42.45 ± 6.15	39.39 ± 8.68	2.51	0.0124*
Day 3	45.85 ± 5.73	39.56 ± 9.91	4.47	0.0001*
Day 7	44.47 ± 8.19	37.04 ± 9.32	5.39	0.0001*
Plateletcrit				
Day 1	0.20 ± 0.09	0.57 ± 1.89	1.43	0.15
Day 3	0.20 ± 0.10	0.23 ± 0.13	1.14	0.25
Day 7	0.14 ± 0.09	0.23 ± 0.14	4.46	0.0001*

Among the platelet indices, the MPV and P-LCR significantly predicted the disease severity on days 1, 3, and 7, PDW showed significant predictive value on days 3 and 7, while PCT was a significant predictor only on day 7. 

Correlation of platelet indices with SOFA and APACHE II scores 

The correlation between platelet indices and SOFA and APACHE II scores was analyzed. MPV was found to be positively correlated with both SOFA and APACHE II scores on days 1 and day 7. However, on day 3, MPV showed a negative correlation with SOFA (r = 0.07) and APACHE II (r = 0.045), though these correlations were not statistically significant (Table 5).

**Table 5:** Correlation between platelet indices (MPV, PDW, P-LCR, and PCT) and SOFA and APACHE II scores on days 1, 3, and 7. MPV: mean platelet volume, PDW: platelet distribution width, P-LCR: platelet large cell ratio, PCT: plateletcrit. *Significant. **Highly significant.

Days	Correlation of platelet indices	SOFA	APACHE II
Day 1 MPV	Pearson correlation coefficient (r)	0.100	0.081
	p-value	0.074	0.150
Day 3 MPV	Pearson correlation coefficient (r)	-0.070	-0.045
	p-value	0.212	0.419
Day 7 MPV	Pearson correlation coefficient (r)	0.008	0.008
	p-value	0.888	0.884
Day 1 PDW	Pearson correlation coefficient (r)	-0.016	-0.027
	p-value	0.769	0.630
Day 3 PDW	Pearson correlation coefficient (r)	-0.260^**^	-0.061
	p-value	0.001	0.273
Day 7 PDW	Pearson correlation coefficient (r)	-0.118^*^	0.026
	p-value	0.035	0.637
Day 1 P-LCR	Pearson correlation coefficient (r)	0.008	-0.155^**^
	p-value	0.889	0.005
Day 3 P-LCR	Pearson correlation coefficient (r)	0.180^**^	0.190^**^
	p-value	0.001	0.001
Day 7 P-LCR	Pearson correlation coefficient (r)	0.293^**^	0.283^**^
	p-value	0.001	0.001
Day 1 PCT	Pearson correlation coefficient (r)	0.083	-0.104
	p-value	0.140	0.063
Day 3 PCT	Pearson correlation coefficient (r)	0.110	0.072
	p-value	0.050	0.200
Day 7 PCT	Pearson correlation coefficient (r)	0.159^**^	0.115^*^
	p-value	0.004	0.041

On day 7, PDW was positively correlated with APACHE II and showed a significant correlation with SOFA scores on days 3 (p = 0.001) and 7 (p = 0.035). Elevated PDW levels were associated with higher SOFA and APACHE II scores.

PDW reflects the variation in platelet size: a higher PDW value indicates greater size variability and increased platelet activation.

P-LCR was positively correlated with SOFA and APACHE II scores on days 3 and 7, with significantly comparable associations (p = 0.001). PCT also showed significant correlations with SOFA (p = 0.004) and APACHE II (p = 0.041) scores on day 7.

Elevated levels of P-LCR and PCT were associated with higher SOFA and APACHE II scores. 

## Discussion

Sepsis remains a major global health concern, with high mortality even after survival, largely due to cardiovascular events and secondary infections. Early recognition and timely intervention are therefore critical. While CRP is more cost-effective than biomarkers such as serum procalcitonin, serum lactate, presepsin (sCD14), and interleukin-6, these markers, though useful for early diagnosis and prognostication, have been reported by several authors [6] to be individually insufficient for reliably predicting disease severity and mortality. Platelet indices, which are readily available as part of a CBC, offer an additional, accessible indicator of systemic inflammation, similar to CRP. Given their basis in platelet size and mass variation, platelet indices were evaluated in correlation with SOFA and APACHE II scores for predicting sepsis severity.

A total of 320 patients were enrolled and analyzed. The incidence of sepsis was evenly distributed across all age groups, with a mean age of 55.17 years. A slightly lower incidence was observed among young adults aged 21-30 years, possibly due to the relative absence of comorbidities. Todi et al. studied 5478 patients in India and reported a mean age of 54.9 years in their epidemiological study of sepsis, which matches the findings of the current study [11]. Sepsis was more commonly observed in male patients, consistent with findings by Gupta and Gupta [12], probably due to increased occupational exposure to infectious pathogens. Sakr et al. and Chatterjee et al. reported a higher mortality rate in men compared to women within the sepsis population [13,14]. In our study, the mortality rate among septic patients was 16.6%, which is comparable to global ICU mortality rates (25.8%) and those reported in India (56%) [15].

In the diagnosis and prognostication of sepsis outcomes, SOFA scores are most commonly used in our ICUs. The results revealed a positive correlation between SOFA scores and mortality among sepsis patients. This finding is supported by Lakhani et al., who concluded that the SOFA score is a useful predictor of mortality in sepsis [15]. The APACHE II score was also used to assess sepsis severity and prognosis in the ICU. The mean APACHE II score positively correlates with the mortality of sepsis; however, it was not statistically significant. This observation aligns with findings by Pandya et al., who also reported limited predictive value of APACHE II for mortality. Similarly, Anjana et al., in a study of 87 patients, found that while APACHE II was reliable for diagnosing sepsis on admission, it was not effective for predicting mortality outcomes [16,17]. In contrast, Thakur et al. concluded that the SOFA scoring system was superior to APACHE II in predicting sepsis outcomes [18].

Association of platelet indices with sepsis outcome (death and discharge)

Platelets play a central role in inflammation and undergo dynamic changes during the inflammatory process. In this study, platelet indices, such as MPV, PDW, P-LCR, and PCT, were analyzed. MPV increases with platelet volume, and in this study, MPV values were greater than 11 fL in non-survivors compared to discharged cases on days 1 and 7. There was a significant correlation between elevated MPV and mortality in sepsis on days 1 (p < 0.0001), 3 (p = 0.0058), and 7 (p = 0.0001). Choudhary et al. reported similar findings in a study of 100 patients in the NICU in New Delhi, India [19]. Likewise, Kim et al., in a study of 345 patients in Italy, observed that MPV increased significantly during the first 72 hours in sepsis non-survivors [20]. The findings of our study are consistent with these earlier reports. 

We found that PDW was positively correlated with death outcomes, as determined by the APACHE II score, and was statistically significant on day 7. PDW measures the variation in platelet sizes, and higher PDW values indicate greater variation in platelet size and increased platelet activation, which may predict more severe illness. These findings are consistent with previous studies conducted by Guclu et al. and Mangalesh et al., who examined 97 patients in North India and found elevated PDW and MPV levels among non-survivors of sepsis [21,22]. The results of our study align with these earlier findings.

In our study, P-LCR was significantly associated with mortality in sepsis on days 1 (p = 0.0124), 3 (p = 0.0001), and 7 (p = 0.0001). These findings are consistent with studies conducted by Gao et al. on 124 patients and Madani et al. on 20 neonates [23,24].

In the present study, PCT was also associated with mortality in sepsis on days 1, 3, and 7. However, the correlation was statistically significant only on day 7, which aligns with the results of Samuel et al., who conducted a study on 170 patients in Karnataka, India [25].

Correlation between platelet indices and SOFA and APACHE II scores

A negative correlation was observed between platelet indices and the SOFA score on day 1 of admission among sepsis patients in the present study. The negative correlation between PDW and SOFA and APACHE II scores suggests that lower PDW levels are associated with higher SOFA and APACHE II scores on days 1 and 3. In contrast, Vardon Bounes et al. observed a significant correlation between MPV and SOFA scores in their study of 300-345 subjects [26]. On day 3, platelet indices like PCT and PDW showed significant correlations with the SOFA score. Elevated PCT levels were associated with higher SOFA and APACHE II scores on day 7. Lower PDW values were associated with higher SOFA scores, consistent with the negative correlation observed on day 3 of sepsis. However, MPV and P-LCR did not show significant correlations with the SOFA score on day 3. Among all the platelet indices, only PDW showed a significant correlation with the APACHE II score on day 3. These findings are consistent with the results of Samuel et al. [25].

On day 7, platelet indices PCT, PDW, and P-LCR showed a significant correlation with the severity of the SOFA score; however, their correlation with the APACHE II score on day 7 was equivocal. There are limited studies investigating the serial correlations between platelet indices and the SOFA and APACHE II scoring systems.

One major limitation of this study is the small sample size, which was constrained by time and limited to a single-center setting. This limitation affects the generalizability of the findings across different subgroups. Given the promising correlations between platelet indices and predictive scores, future studies with larger sample sizes and multicentric designs are recommended to better assess the predictive value of hematological parameters for sepsis outcomes.

## Conclusions

The present study concluded that sepsis outcomes were significantly associated with age and the APACHE II score at the time of admission. Among the platelet indices, PDW and PCT showed significant correlations with both the SOFA and APACHE II scores on days 3 and 7. Elevated PDW and PCT values have the potential to predict mortality, similar to the SOFA and APACHE II scores. Additionally, P-LCR on day 7 was found to be an effective predictor of sepsis outcomes. These findings suggest that platelet indices may serve as valuable prognostic markers for sepsis outcomes, particularly in resource-poor settings, where they can be used as an alternative to SOFA and APACHE II scores. To further validate and establish the role of platelet indices in predicting sepsis outcomes, multicentric studies involving larger patient populations are needed.
